# PF-CMNet: Progressive Frequency-Aware Cross-Modal Network with Missing-Modality Distillation for 3D Brain Tumor Segmentation

**DOI:** 10.3390/brainsci16060588

**Published:** 2026-05-29

**Authors:** Haokun Wang, Shuyi Wang, Yuqi Li, Xinrong Miao, Chenyi Cao

**Affiliations:** School of Health Science and Engineering, University of Shanghai for Science and Technology, Shanghai 200093, China; 243352425@st.usst.edu.cn (H.W.); 242322224@st.usst.edu.cn (Y.L.); 243352327@st.usst.edu.cn (X.M.); 255372534@st.usst.edu.cn (C.C.)

**Keywords:** multimodal MRI, missing modality, brain tumor segmentation, cross-modal learning, knowledge distillation

## Abstract

**Highlights:**

**What are the main findings?**

- PF-CMNet integrates frequency-aware cross-modal representation learning, progressive detail-oriented decoding, and teacher–student distillation for robust 3D multimodal brain tumor segmentation.
- Experiments on MSD Task01_BrainTumour and BraTS2021 show that PF-CMNet achieves competitive segmentation performance under full-modality conditions and maintains stronger robustness when MRI modalities are missing.

**What are the implications of the main findings?**

- The results suggest that explicitly modeling complementary information across MRI modalities can improve tumor subregion segmentation, particularly for complex boundaries and small enhancing tumor regions.
- The proposed missing-modality distillation strategy provides a practical framework for improving segmentation stability in clinical scenarios where complete multimodal MRI acquisition may not always be available.

**Simple Summary:**

Accurate brain tumor segmentation from multimodal magnetic resonance imaging is important for neurosurgical planning and image-guided procedures. However, tumor boundaries are often unclear, small enhancing tumor regions are difficult to identify, and some imaging sequences may be unavailable in clinical practice. To address these challenges, this study proposes a three-dimensional brain tumor segmentation network that learns complementary information from different imaging sequences and improves robustness when one or more sequences are missing. The proposed method combines frequency-aware cross-modal feature learning, progressive boundary detail recovery, and teacher–student distillation under missing-modality conditions. Experiments on public brain tumor datasets showed that the method achieved competitive segmentation accuracy and maintained more stable performance when imaging sequences were missing. These findings suggest that the proposed framework may provide a useful technical basis for automated brain tumor analysis, while further multicenter and workflow-level validation is still required before clinical deployment.

**Abstract:**

Background/Objectives: Accurate automatic segmentation of multimodal magnetic resonance imaging (MRI) is essential for neurosurgical planning and image-guided procedures. However, existing three-dimensional segmentation models often struggle with low lesion-to-tissue contrast, ambiguous tumor boundaries, small enhancing tumor regions, and performance degradation caused by missing imaging modalities. This study aimed to develop a robust segmentation framework that improves cross-modal representation learning, boundary recovery, and segmentation performance under incomplete-input conditions. Methods: We propose PF-CMNet, a Progressive Frequency-Aware Cross-Modal Network with Missing-Modality Distillation for three-dimensional brain tumor segmentation. The network introduces a Cross-Modal Selective Frequency Attention module in the early encoder stage to model modality-specific frequency responses and spatially adaptive cross-modal correlations. A Progressive Cross-Scale Detail Fusion decoder is further employed to aggregate multilevel semantic features and refine high-resolution boundary details. To enhance robustness under missing-modality conditions, a teacher–student distillation strategy transfers full-modality predictions and shallow feature knowledge to a student network trained with random modality dropout. Results: On the MSD Task01_BrainTumour dataset, PF-CMNet achieved an average Dice score of 84.3%, with Dice scores of 79.6%, 82.8%, and 90.4% for enhancing tumor, tumor core, and whole tumor, respectively. On the BraTS2021 dataset, the model achieved an average Dice score of 88.2% and the lowest average 95th percentile Hausdorff distance among the compared methods. In predefined complete-modality absence stress tests, where unavailable MRI sequences were zero-masked to model the absence of input modalities rather than partial image degradation, the distilled model maintained average Dice scores of 78.64%, 82.58%, 58.39%, 82.03%, and 79.29% when FLAIR, T1, T1ce, T2, and T1 + T2 were unavailable, respectively. Conclusions: PF-CMNet provides a unified framework for multimodal brain tumor segmentation, improving full-modality segmentation accuracy, boundary consistency, and robustness to incomplete MRI inputs while maintaining a favorable accuracy–efficiency trade-off.

## 1. Introduction

Brain tumors are among the diseases with the highest mortality and disability rates worldwide [1]. Among them, gliomas pose significant challenges for clinical treatment due to their highly invasive nature and indistinct borders [2]. Precise, automated segmentation of brain tumors is a critical component of preoperative planning in neurosurgery and the deployment of mixed reality (MR) surgical navigation systems [3,4,5]. In these intelligent navigation scenarios, which are increasingly shifting toward contactless voice interaction, the three-dimensional hologram generated from the segmented reconstruction is superimposed directly onto the patient’s actual anatomical structures [6,7]. Therefore, accurate region delineation and reliable boundary reconstruction are more clinically relevant than pursuing parameter reduction alone [8]. Multimodal MRI is the primary non-invasive imaging modality for glioma assessment, as different MRI sequences provide complementary information about lesion morphology and tissue characteristics [9,10]. The FLAIR sequence is more sensitive to edematous areas, the T1ce sequence is better suited for visualizing enhancing tumor regions, while T1 and T2 sequences provide fundamental anatomical structures and soft tissue contrast information [11,12]. Combining information from multi-modal sequences facilitates a more comprehensive understanding of the tumor’s spatial characteristics and boundary morphology [13]. However, brain tumors exhibit significant heterogeneity in morphology, boundaries, and internal structure [11]. In addition, missing modalities frequently occur in clinical acquisition because of limited scanning time, protocol variations, patient motion, or insufficient image quality [14,15]. When deploying automatic segmentation models, effectively modeling cross-modal complementary information while maintaining robustness in scenarios where certain modalities are missing remains a critical challenge [16].

In recent years, Convolutional Neural Networks (CNNs) [17,18,19] and Transformers [20,21,22,23] have successively emerged as primary architectures in medical image segmentation research, significantly advancing the development of 3D brain tumor automatic segmentation [24]. Early methods such as U-Net [17], 3D U-Net [18], and V-Net [19] established the fundamental paradigm for volumetric segmentation through encoder–decoder and skip-connection architectures, demonstrating stability in multi-scale local feature extraction via local receptive fields. However, convolutional architectures still have limitations in modeling long-range dependencies [20]. To address this, Transformer-based models or hybrid architectures—such as UNETR [21], Swin UNETR [25], nnFormer [23], and SegFormer3D [26]—have been introduced into the field of medical image segmentation. Through hierarchical feature extraction and global context modeling, these models have demonstrated superior performance in complex tumor segmentation tasks, suggesting the importance of global modeling capabilities for understanding complex lesion boundaries and long-range context [21,23,25,26].

In multimodal brain tumor segmentation, a common strategy is to stitch together different MRI sequences at the input layer [27,28]. Although these models can implicitly fuse multimodal information at deep layers, they lack explicit modeling of early-stage cross-modal interactions [29]. Because different MRI modalities emphasize different tumor subregions, simple shared encoding may fail to fully exploit modality-specific cues for edema, tumor core, and enhancing tumor regions [11,12].

Furthermore, although lightweight decoders are effective for reducing computational cost, their simplified reconstruction pathways may be insufficient for recovering fine-grained structures in challenging 3D brain tumor segmentation, especially for small enhancing tumor regions and irregular tumor boundaries [26,30,31]. In smaller areas of tumor enhancement, the limitations of such models may be more pronounced, as these regions typically exhibit poor contrast, limited volume, and irregular shapes, making accurate delineation particularly challenging [8].

In addition to these architectural challenges, most existing state-of-the-art (SOTA) models are trained and inferred under the condition of complete four-modal data input. In actual clinical scanning environments, missing modalities are common due to patient conditions, limited scanning time, protocol variations, equipment constraints, and motion artifacts [14,15]. Models trained solely under complete modal conditions are often highly dependent on the completeness of the input modalities, leading to unstable performance during actual deployment. Existing methods for training with missing modalities primarily aim to improve segmentation performance through modal completion or robust fusion; however, the modality-dependent frequency characteristics and their potential influence on boundary-sensitive representation learning remain less explicitly explored [15,32,33,34]. For brain tumor segmentation, the absence of specific modalities not only weakens spatial semantic complementarity but may also result in insufficient representation of boundary and texture information [12,35]. Therefore, spatial-domain fusion alone may be insufficient to preserve boundary-sensitive and modality-specific cues under incomplete-input conditions.

Recent hybrid physics–AI studies, such as the hybrid FEM-AI framework proposed by Pratticò et al. for thermographic monitoring of biomedical electronic devices, have shown that biomedical AI representations can be better interpreted when related to measurable physical or signal-domain patterns [36]. Inspired by this methodological perspective, our frequency-aware design introduces a structured spatial-frequency representation for multimodal MRI segmentation, while not claiming that the selected frequency bands constitute a complete physical model of MRI tissue contrast.

Based on the above analysis, we consider that missing modalities may affect not only high-level semantic representation but also low-level boundary-sensitive cues that are reflected in modality-dependent frequency patterns. To address this issue, we introduce an early frequency-aware cross-modal interaction module to model band-specific modality responses before deep semantic abstraction. Based on this representation, we further develop a teacher–student distillation strategy to transfer full-modal knowledge to scenarios with incomplete input. Additionally, we design a progressive cross-scale detail fusion decoder aimed at preserving and recovering high-resolution details from frequency-enhanced shallow features.

To this end, this study proposes PF-CMNet, a Progressive Frequency-Aware Cross-Modal Network with Missing-Modality Distillation, for 3D multimodal brain tumor segmentation. On the encoding side, a cross-modal selective frequency-domain attention module is employed to enhance shallow-layer modal interaction; on the decoding side, progressive cross-scale detail fusion improves boundary recovery. During training, a two-stage optimization strategy based on residual control is combined with teacher–student-style missing-modality distillation, enabling the model to maintain its full-modal segmentation capability while further enhancing stability and robustness under incomplete input conditions.

The main contributions of this study are summarized as follows:

- We propose a Cross-Modal Selective Frequency Attention module (CMSFA) that explicitly models cross-modal complementary relationships in the early stages of the encoder. By leveraging band-specific statistics, cross-modal gain modulation, and spatial gating, it enhances shallow discriminative features, addressing the shortcomings of traditional input concatenation in early cross-modal interaction.
- We design a Progressive Cross-Scale Detail Fusion Decoder (PCDF) to convert frequency-enhanced shallow-layer information into boundary-sensitive segmentation outputs. By combining top-down semantic fusion with high-resolution detail enhancement, this decoder improves the reconstruction of subtly enhancing tumor regions and complex tumor boundaries.
- We construct Teacher–Student Distillation for Missing-Modality Scenarios (TSD-MS) that transfers full-modality prediction and early feature knowledge to a student network trained with random modality dropout, thereby improving segmentation stability under incomplete MRI inputs.

## 2. Related Work

### 2.1. Transformer-Based Medical Image Segmentation Methods

To address the limitations of convolutional neural networks in modeling long-range dependencies, the Transformer architecture has been widely adopted in the field of medical image segmentation in recent years [20,21,23,25,37]. Unlike traditional convolutional operators, which primarily rely on local receptive fields, the self-attention mechanism can establish global connections between voxels over a larger range, thereby demonstrating superior performance in tasks involving complex structures and large-scale global context modeling [38]. UNETR represents volumetric images as patch embeddings and uses a Transformer encoder to extract global contextual information and combines this with a U-shaped decoding path to perform voxel-level predictions, thereby validating the effectiveness of pure Transformer encoding in 3D medical segmentation [21]. TransBTS adopts a hybrid architecture combining 3D CNNs with Transformers, introducing self-attention mechanisms to convolutional feature encoding to enhance the modeling of global semantic relationships [37].

To reduce the computational burden of Transformer models on high-dimensional volumetric data, researchers have further proposed hierarchical Transformer designs better suited for high-dimensional medical volume data. Swin UNETR introduces window-based self-attention with shifted windows, reducing the computational cost of attention while enabling cross-window information exchange in volumetric segmentation [25]. nnFormer alternates between convolutional and Transformer layers and introduces multi-scale volume attention and skip-connection attention to balance local details with global semantic representation [23].

Meanwhile, lightweight Transformer segmentation frameworks have also garnered increasing attention. SegFormer achieves a balance of efficiency and accuracy in semantic segmentation through a position-free hierarchical Transformer encoder and a lightweight decoder [39]; SegFormer3D extends this design to volumetric medical image segmentation by employing a four-stage hierarchical Transformer encoder and an All-MLP decoder for efficient multi-scale feature fusion [26]. Although Transformer-based segmentation models have improved global context modeling and multi-scale representation learning, most designs primarily focus on encoder-side semantic modeling, while early cross-modal interaction, decoder-side detail recovery, and robustness to incomplete multimodal inputs are less jointly investigated within an efficient 3D framework.

### 2.2. Cross-Modal Fusion in Multimodal MRI Segmentation

In multimodal MRI brain tumor segmentation, different modalities exhibit distinct response characteristics to lesion subregions. For example, FLAIR is sensitive to peritumoral edema, T1ce highlights contrast-enhanced tumor regions, and T1/T2 provide complementary anatomical and tissue-contrast information [9,10]. The differences in how different modalities respond to tumor subregions determine that the core of cross-modal modeling is not a simple concatenation of input information, but rather the selection of valuable modality information during the feature extraction process.

Most existing cross-modal modeling methods employ simple channel concatenation of multi-modal images, mapping different modality information into a shared space before performing unified modeling via convolutional and attention modules [15,29,32,33]. While such methods are relatively simple to train, they struggle to fully integrate the deep correlations between modalities.

Another class of methods further incorporates attention mechanisms with region-aware fusion or modal alignment measures to enhance dynamic interaction capabilities between modalities. HeMIS learns modality-specific latent representations and aggregates the available modalities using mean and variance statistics, providing an early framework for hetero-modal segmentation [15]. RFNet utilizes region-aware fusion to enhance modal selectivity across different tumor subregions, showing the importance of spatially adaptive cross-modal fusion [32]. mmFormer introduces modal-specific encoders and a cross-modal Transformer to establish global long-range correlations within each modality [29]. CMAF-Net further explores attention-based cross-modal fusion by adaptively weighting multimodal features, improving feature integration at the spatial representation level [40]. These studies demonstrate that explicitly modeling modality availability, regional relevance, and inter-modal dependencies is beneficial for multimodal brain tumor segmentation.

Existing methods primarily focus on feature alignment and interaction between different modalities in the spatial domain. However, spatial-domain fusion alone may not explicitly characterize how different modalities contribute to low-frequency anatomical context, mid-frequency boundary transitions, and high-frequency texture variations. In contrast, differences in frequency responses across modalities provide an alternative perspective for characterizing such complementary information, yet this aspect remains underexplored in current studies [29,33].

### 2.3. Decoder Design for Detail Recovery

In 3D medical image segmentation, the design of the decoder must balance segmentation accuracy with computational cost and the number of parameters.

Early U-shaped networks were widely adopted due to their simple and efficient encoder–decoder architecture. Among these, U-Net established the fundamental paradigm for multi-scale feature reconstruction by restoring spatial resolution through skip connections and progressive upsampling [17]. Building on this foundation, UNet++ introduced nested skip connections to bridge the semantic gap between shallow-level details and deep-level semantics, further enhancing the multi-scale feature fusion capability during the decoding stage [30].

In recent years, to control the parameter scale and computational cost of 3D segmentation models, lightweight decoder design has gradually become a key direction in medical image segmentation. SegFormer3D performs reconstruction using multiscale features output by a hierarchical encoder, achieving good segmentation performance with low computational cost and computational load [26]. However, when faced with the segmentation of complex and small-volume 3D brain tumors, there is an issue of insufficient detail recovery.

To enhance the decoder’s multi-scale recovery capabilities, recent studies have begun improving decoders from the perspectives of multi-scale attention and structure-aware mechanisms. EMCAD introduces an efficient multi-scale convolutional attention decoder that integrates complex spatial relationships through multi-scale depth-wise convolutions and channel, spatial, and batch-wise gated attention, achieving a good balance between performance and computational efficiency [31]; however, its design is primarily oriented toward 2D medical image segmentation. Furthermore, some lightweight methods, such as LETNet [41] and LightM-UNet [42], have reduced the number of parameters and inference overhead through simplified encoder–decoder architectures, dilated convolutions, or lightweight upsampling modules. However, these methods are primarily designed for 2D or single-modal scenarios, and their support for cross-modal collaboration and complex boundary recovery in multi-modal 3D brain tumor segmentation remains limited. When transferred to multimodal 3D brain tumor segmentation, these lightweight designs often struggle to simultaneously balance computational efficiency and complex boundary recovery.

### 2.4. Missing-Modality Robustness in Multimodal Segmentation

Missing MRI modalities are a common challenge in clinical imaging and retrospective multimodal brain tumor analysis [16,43]. Existing missing-modality learning methods can be broadly categorized into three types: modality completion, robust feature fusion, and knowledge distillation or transfer-based strategies. One line of research attempts to use GAN methods [44,45] to synthesize missing MRI sequences; artifacts in synthesized modalities may propagate to the segmentation stage and introduce additional uncertainty. Another line of work avoids explicit synthesis and instead learns modality-robust representations. HeMIS maps each modality to a common space and aggregates different modalities using mean and variance statistics [15]. Recent studies such as mmFormer and M2FTrans have introduced modality-specific encoders and multi-head attention mechanisms to adaptively weight spatial and channel-based modalities, thereby enhancing robustness for missing modalities [29,33]. Despite these advances, maintaining stable image characterization becomes more challenging in the absence of multiple information-rich imaging modalities, particularly for small areas of enhancing tumors that rely heavily on T1ce contrast information.

In addition to robust fusion, knowledge distillation has been explored in recent years for missing-modality segmentation, aiming to transfer full-modality prior knowledge across networks [34,46,47]. SMU-Net first distills semantic information from the complete-modality branch into the missing-modality path, validating the effectiveness of the teacher–student paradigm in incomplete MRI segmentation [34]. Subsequently, KD-Net transfers full-modality knowledge to the missing-modality branch by focusing on aligning the probability distributions of the teacher and student models [48]. TASCCNet further integrates multi-scale Transformer distillation, dual-mode logit distillation, and global style matching, achieving progress in boundary recovery and representation transfer [47]. Although knowledge distillation has demonstrated great potential, existing frameworks often focus on feature transfer at the pure prediction layer or deep semantic levels, neglecting the preservation of shallow cross-modal heterogeneous features during the early encoding stages.

Robustness under incomplete or degraded biomedical inputs is not limited to multimodal MRI segmentation. Recent biomedical monitoring studies have also emphasized the importance of instrumental validation and signal stability assessment under noisy and non-ideal conditions. For example, a smart electronic device-based monitoring system was developed to monitor specific absorption rate (SAR) and temperature variations during indoor human–tissue interaction. This system used sensor-based acquisition, cloud transmission, and feedforward neural-network analysis to evaluate signal variations under both stationary and moving conditions [49]. Although the application scenario differs from multimodal MRI segmentation, this study highlights a shared methodological concern: robustness evaluation should distinguish performance under ideal input conditions from performance under incomplete, noisy, or degraded real-world inputs. Therefore, in the present study, the zero-masked missing-modality experiments are interpreted as complete-modality absence stress tests rather than as a substitute for comprehensive artifact- or noise-degradation validation.

Compared with existing methods, this work distinguishes itself by not limiting the improvement to a single module. Instead, within a 3D Transformer-based segmentation framework, the proposed method jointly addresses three key issues: early cross-modal frequency-domain modeling, decoder-side detail recovery, and knowledge distillation under missing-modality conditions. This design aims to maintain competitive full-modality segmentation performance while improving stability and robustness under incomplete-input conditions, and to achieve a favorable balance between segmentation accuracy and computational cost.

## 3. Method

### 3.1. Overview of Network Architecture

**Figure 1:**
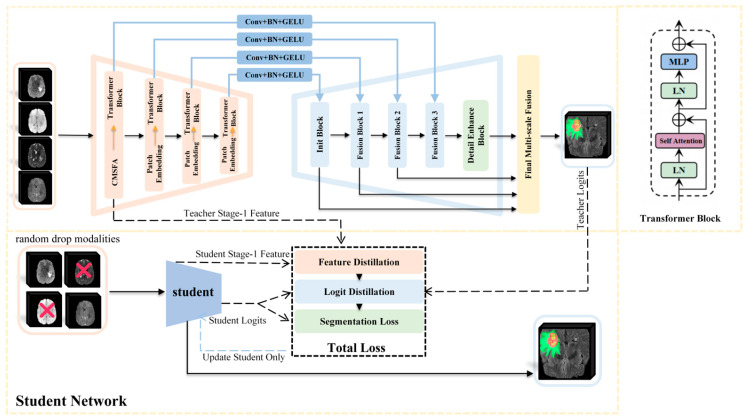
Overall architecture of PF-CMNet.

PF-CMNet adopts an encoder–decoder architecture that integrates frequency-aware cross-modal representation learning, progressive cross-scale detail fusion, and missing-modality distillation into a unified framework for 3D brain tumor segmentation. The overall architecture of PF-CMNet is illustrated in Figure 1.

Given an input four-modal 3D MRI image $X\in R^{B\times M\times D\times H\times W}$, where *B* is the batch size, *M* = 4 represents the four MRI modalities (FLAIR, T1, T1ce, and T2), and *D*, *H*, and W denote the depth, height, and width of the input data, respectively. The network first extracts multiscale features through a four-stage hierarchical encoder. In the first stage of the encoder, a cross-modal selective frequency attention module (CMSFA) is introduced to enhance shallow-level cross-modal interaction. Subsequently, the encoder performs layer-wise downsampling operations in the remaining stages to extract higher-level, multi-scale contextual features. The decoding stage employs a Progressive Cross-Scale Detail Fusion (PCDF) module to perform top-down, hierarchical fusion and reconstruction of feature information from different stages of the encoder, thereby outputting the final 3D segmentation results.

To enhance robustness under incomplete input conditions, PF-CMNet further employs a teacher–student missing-modality distillation strategy. Using PF-CMNet under full-modality conditions as the teacher model, the student network inherits the same network architecture as the teacher model. Dual constraints are imposed on both the feature and prediction layers of the student network under random missing-modality scenarios, enabling the student network to inherit the teacher’s discriminative knowledge and cross-modal representation capacity under incomplete inputs.

### 3.2. Cross-Modal Selective Frequency Attention

**Figure 2:**
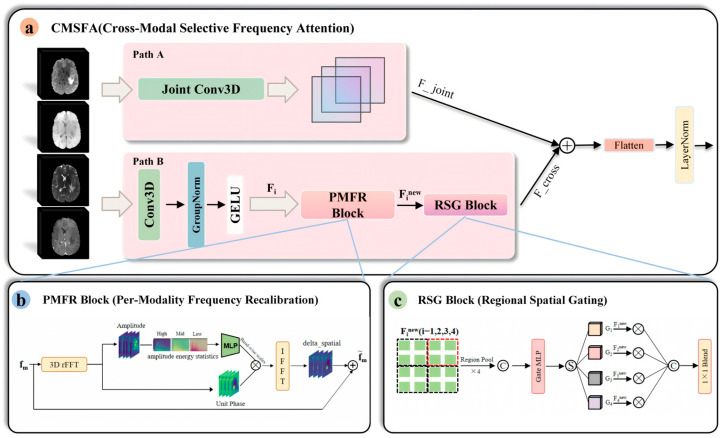
Structure of the CMSFA module.

Different MRI modalities focus on distinct tumor-related structures; if they are simply combined as input channels, modality-specific low-level information may be masked during the initial feature extraction process. To preserve and selectively enhance this information, CMSFA introduces a dual-path design that combines a stable spatial fusion branch with a modality-based frequency enhancement branch, as shown in Figure 2.

Path A is a baseline spatial joint branch that preserves stable low-level representations. It directly applies a 3D convolution with a stride of 4 and a size of 7 × 7 × 7 to the multimodal input for joint feature extraction, generating basic local spatial features:

(1) $$F_{joint}=H_{conv3d}(X,W_{joint})\in R^{B\times C\times D^{\prime }\times H^{\prime }\times W^{\prime }}$$

Here, *C* represents the embedding dimension, while $D^{\prime }$, $H^{\prime }$, and $W^{\prime }$ denote the spatial dimensions after downsampling. This path is designated as Path A to ensure stable input and preserve a stable representation of the original input.

Path B performs modality-wise frequency-domain enhancement. We process the input $X_{m}\in R^{B\times 1\times D\times H\times W}$ for each modality using a separate per-modality embedding block to generate a modality-specific representation $f_{m}\in R^{B\times C\times D^{\prime }\times H^{\prime }\times W^{\prime }}$. Subsequently, we perform a real-valued 3D fast Fourier transform (3D rFFT) on $f_{m}$:

(2) $$Z_{m}=F(f_{m})=A_{m}⊙e^{j\Phi_{m}}$$

Here, $A_{m}(u,v,w)$ represents the amplitude spectrum and $\Phi_{m}(u,v,w)$ represents the phase spectrum. To establish an interpretable and structured modulation in the frequency domain and facilitate the calculation of the Euclidean distance from each point in the three-dimensional frequency grid to the zero-frequency component, the spectrum is divided into K non-overlapping frequency bands, with K set to 3 to correspond to the low-, mid-, and high-frequency ranges. The number of frequency bands was set to K = 3 to provide a compact coarse-to-fine decomposition of spatial-frequency information. This setting was selected as a lightweight and interpretable design rather than as a theoretically optimal hyperparameter. Low-frequency components are generally associated with coarse spatial intensity variations and global anatomical morphology, mid-frequency components may reflect regional transitions and boundary-like structures, and high-frequency components may contain fine local variations, texture details, and noise. A smaller K may provide overly coarse decomposition and fail to distinguish boundary-related transitions from fine local details, whereas a larger K would introduce additional band-wise statistics and modulation parameters and may increase sensitivity to high-frequency noise. Therefore, K = 3 was adopted to balance representational granularity. To capture the global response intensity of different modalities across frequency components, the normalized energy statistic for the *m*-th modality in the k-th frequency band is calculated as follows:

(3) $$e_{m,k,c}=\frac{\sum_{u,v,w}\left(A_{m,c}(u,v,w)⊙\Omega_{k}(u,v,w)\right)}{\sum_{u,v,w}\Omega_{k}(u,v,w)+\epsilon }$$

To prevent the denominator from becoming zero in the case of extremely small constants, we set ϵ to $10^{-6}$ and $\Omega_{k}(u,v,w)\in \{0,1\}$ to the corresponding binary mask generated. The statistics from all modalities and frequency bands are concatenated to form a vector $e\in R^{B\times (M\cdot K\cdot C)}$. We then feed e into a cross-modal multi-layer perceptron (MLP) to generate the adaptive scaling tensor $S\in R^{B\times M\times K\times C}$:

(4) $$S=1.0+0.3\cdot \tanh({MLP}_{cross}(e))$$

To bound the frequency-domain modulation gain and suppress gradient instability caused by excessive amplification of high-frequency noise, the hyperbolic tangent function tanh(⋅) is introduced. Furthermore, to prevent excessive frequency fluctuations from destabilizing the feature distribution during the early training stages, a conservative regularization factor of 0.3 was adopted. By restricting the adaptive gain to the range [0.7, 1.3], a balance was achieved between sufficient cross-modal enhancement and maintaining a stable baseline representation.

Let $S_{m,k,c}$ denote the scalar scaling factor corresponding to the *m*-th modality, *k*-th frequency band, and *c*-th channel in this tensor. After broadcasting it along the spatial frequency dimension, the amplitude spectrum of a specific frequency band is modulated independently:

(5) $$△Z_{m,c}=\sum_{k=1}^{K}(S_{m,k,c}-1.0)\cdot A_{m,c}⊙\Omega_{k}⊙e^{j\Phi_{m,c}}$$

While preserving the original phase spectrum, the $\Delta Z_{m,c}$ values from all channels are concatenated to obtain the frequency-domain residual tensor Δ$Z_{m}\in C^{C\times D\prime \times H\prime \times W\prime }$. This is then mapped back to the spatial domain using a three-dimensional inverse fast Fourier transform (3D iRFFT) to obtain the frequency-enhanced feature:

(6) $${\tilde{f}}_{m}=f_{m}+F^{-1}(\Delta Z_{m})$$

Relying solely on global frequency-band statistics for modulation can establish frequency-domain selectivity relationships between modalities to some extent, but it may still overlook differences in modality dependence across different local spatial regions. In this study, we designed a spatial gating branch to apply weights to the frequency-domain-enhanced modal features.

We perform three-dimensional adaptive average pooling on ${\tilde{f}}_{m}$ with a kernel size of R = 4 to obtain a coarse-grained regional description. Following mapping through a linear layer and normalization via the Softmax function, we compute the global spatial gating tensor $G\in R^{B\times M\times R\times R\times R}$:

(7) $$G={Softmax}_{m}(MLP(Pool([{\tilde{f}}_{1},\ldots ,{\tilde{f}}_{M}])))$$

Let $G_{m}\in R^{B\times 1\times R\times R\times R}$, which is extracted by slicing the global spatial gating tensor G along the modality dimension *M*, serve as the submask for the *m*-th modality. After extracting the modality-specific slice and upsampling it to the original feature scale via trilinear interpolation, we perform element-wise multiplication with ${\tilde{f}}_{m}$ to achieve weighted concatenation and fusion. Finally, we reduce the dimension and fuse the result into $F_{cross}$ via a 1 × 1 × 1 convolution, where

(8) $$F_{cross}=Conv_{1\times 1\times 1}\left(Concat{\left(G^{\left(m\right)}⊙{\tilde{f}}_{m}\right)}_{m=1}^{M}\right)$$

⊙ denotes element-wise multiplication. The final module output of CMSFA is parameterized by a learnable scalar α to form a residual structure:

(9) $$F_{out}=F_{joint}+\sigma (\alpha )\cdot (F_{cross}-F_{joint})$$

where σ(⋅) is the Sigmoid activation function. The initialization of α was chosen according to the residual-gating role of this parameter. Since the contribution of the CMSFA residual branch is controlled by sigmoid(α), initializing α to 0 would assign an initial residual weight of 0.5, allowing the newly introduced frequency-domain branch to contribute too strongly at the beginning of training. Such an aggressive initialization may disturb the feature distribution of the baseline spatial representation. In contrast, α = −2.0 corresponds to sigmoid(−2.0) ≈ 0.12, which provides a conservative but non-zero residual contribution. This setting allows the frequency-enhanced branch to participate in optimization while preventing it from dominating the original spatial representation during the early training stage. More negative values, such as α = −3.0, would further suppress the residual branch and may slow down its adaptation. Therefore, α = −2.0 was used as a conservative residual-gating initialization rather than as a theoretically optimal hyperparameter. Considering that shallow-layer features possess higher resolution and are closer to the original modal differences, this paper introduces CMSFA only in the initial stage of the encoder to maximize the benefits of early cross-modal complementary modeling while controlling additional complexity. Furthermore, a two-stage optimization strategy is adopted during training to first achieve a smooth alignment between the frequency-domain branch and the baseline representation space, followed by global joint fine-tuning. Although CMSFA introduces explicit frequency-band statistics and spatial gating to improve the transparency of cross-modal feature modulation, these design components should be regarded as structurally interpretable mechanisms rather than direct visual explanations of the model’s final decision process.

### 3.3. Progressive Cross-Scale Detail Fusion Decoder

**Figure 3:**
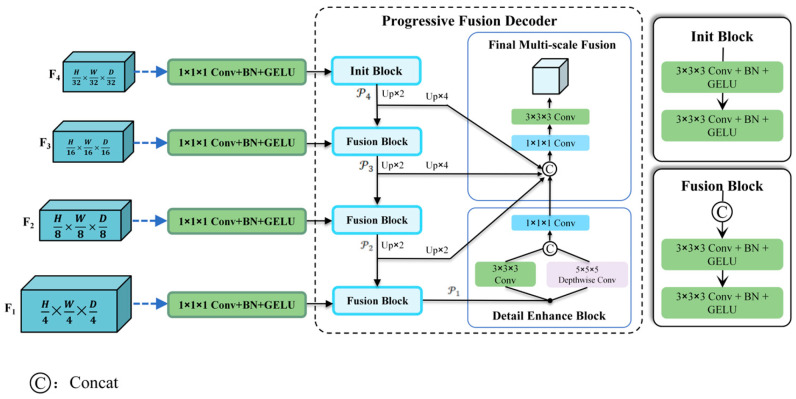
Structure of the PCDF decoder.

CMSFA enhances cross-modal discriminative features in the shallow layers. However, if the lightweight decoder of the original framework is retained, the frequency-enhanced complementary information produced by CMSFA cannot be effectively translated into accurate boundary recovery. Therefore, we propose the Progressive Cross-Scale Detail Fusion Decoder (PCDF). The PCDF architecture is shown in Figure 3.

Let $F_{i}\in R^{B\times C_{1}\times D_{i}\times H_{i}\times W_{i}}$ denote the multi-scale features at the encoder’s output level, where $i\in \left\{1,2,3,4\right\}$. First, by utilizing 1 × 1 × 1 convolutions and batch normalization, all feature levels are uniformly mapped to a channel dimension of $C_{emb}=256$, denoted as $F_{i}^{\prime }$.

The decoder adopts a top-down cascaded structure to fuse multi-scale context (Top-Down Fusion Block). The update process for the intermediate decoding feature $P_{i}$ is defined as follows:

(10) $$P_{4}=\Psi_{refine}(F_{4}^{\prime })$$

(11) $$P_{i}=\Psi_{fuse}\left(\left[{F_{i}}^{\prime },U_{3D}(P_{i+1})\right]\right),\;i\in \{3,2,1\}$$

where $U_{3D}(\cdot )$ denotes 3D trilinear interpolation upsampling, and $\left[\cdot ,\cdot \right]$ denotes channel concatenation. The mapping functions $\Psi_{refine}$ and $\Psi_{fuse}$ are both composed of two consecutive 3 × 3 × 3 Conv-BN-GELU modules, used to smooth features and eliminate upsampling artifacts.

At the highest-resolution $P_{1}$ stage, a detail enhancement branch is introduced to refine boundary-sensitive local structures, especially around small enhancing tumor regions. This branch consists of two parallel paths: Local $B_{local}$ and Global $B_{large}$:

(12) $$B_{local}=\Psi_{3\times 3\times 3}(P_{1})$$

(13) $$B_{large}=\Psi_{1\times 1\times 1}\left(\sigma_{GEU}\left(B_{BN}(W_{5\times 5\times 5}^{DW}ࢩP_{1})\right)\right)$$

Path $B_{local}$ employs standard 3 × 3 × 3 convolutions to capture edge gradient changes; Path $B_{large}$ uses 5 × 5 × 5 large-kernel depthwise 3D convolutions (Depthwise 3D Conv) to expand the local receptive field without significantly increasing parameter complexity, thereby enhancing the modeling capability of continuous lesion contours. After concatenating the two sets of features, channel reorganization is performed via a 1 × 1 × 1 convolution, and the results are fed back to the main feature in a residual manner:

(14) $$P_{1}^{enhanced}=P_{1}+\Psi_{1\times 1}\left(\left[B_{local},B_{large}\right]\right)$$

Finally, features from all scales are uniformly upsampled and concatenated. Through the final feature fusion module Φ(⋅), the segmentation results $\hat{Y}$ with an output dimension of $N_{c}$ = 3 are produced by the prediction head:

(15) $$\hat{Y}={Conv}_{1\times 1\times 1}\left(\Phi \left(\left[P_{1}^{enhanced}],U_{3D}(P_{2}),U_{3D}(P_{3}),U_{3D}(P_{4}\right)\right)\right)$$

Here, Φ(⋅) denotes the final feature aggregation module, consisting of a 1 × 1 × 1 channel fusion convolution followed by a 3 × 3 × 3 convolution for refinement.

Unlike approaches that directly employ heavy decoders, PCDF introduces convolution-based enhancement only at necessary cross-scale fusion stages and the highest resolution stage. This design aims to improve detail reconstruction while maintaining moderate decoder complexity. In this way, PCDF complements CMSFA by converting shallow cross-modal cues and deep semantic features into progressively refined segmentation predictions.

### 3.4. Teacher–Student Distillation for Missing-Modality Scenarios

**Figure 4:**
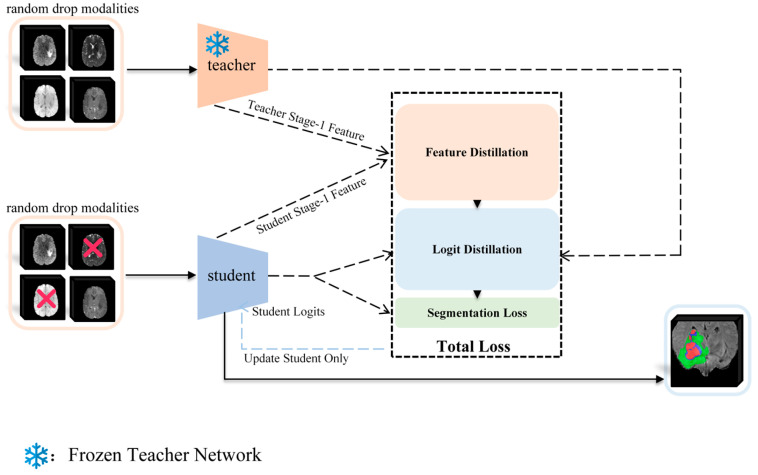
Teacher–student distillation framework for missing-modality learning.

In real-world clinical scans, missing modalities often occur due to objective factors such as differences in acquisition protocols, equipment limitations, or patient conditions [16,43]. To improve the segmentation stability of PF-CMNet under incomplete input conditions, this paper further proposes a Teacher–Student Distillation strategy tailored for missing-modality scenarios (TSD-MS) as shown in Figure 4.

We use a PF-CMNet trained to convergence on full-modality MRI as the teacher network *T* with frozen parameters and then construct a student network *S* with the same backbone architecture. During training, we apply random modality dropout to the student network’s input *x* to simulate modality loss in clinical scenarios. The random sampling strategy is defined as follows: the complete four-modality input is retained with a probability of 25%, one randomly selected modality is masked with a probability of 50%, and two randomly selected modalities are masked with a probability of 25%. This distribution is used as a heuristic rather than an assumed clinical prevalence, aiming to expose the student network to complete, mildly incomplete, and severely incomplete input patterns. It should be noted that random modality dropout and teacher—student distillation play different roles in TSD-MS. Random modality dropout increases the diversity of incomplete-input patterns and prevents the student network from over-relying on a fixed set of modalities. However, dropout alone only changes the input distribution and does not provide an explicit target for recovering the discriminative representation available under full-modality conditions. In contrast, the frozen full-modality teacher provides stable prediction-level and feature-level supervisory signals, encouraging the student network to approximate the teacher’s full-input response even when part of the input information is unavailable. Therefore, in TSD-MS, modality dropout is used to construct incomplete-input learning scenarios, whereas teacher-guided distillation is used to constrain the direction of representation learning under these scenarios. It preserves sufficient full-modality cases for learning strong upper-bound representations, while also increasing exposure to challenging missing-modality settings to improve robustness, thereby enabling the student network to learn stable feature representations under varying levels of information completeness.

(16) $$L_{total}=L_{seg}({\hat{Y}}_{S},Y_{gl})+\lambda_{logit}L_{KD}({\hat{Y}}_{S},{\hat{Y}}_{T})+\lambda_{feat}L_{FD}(F_{S}^{\left(1\right)},F_{T}^{\left(1\right)})$$

Here, $L_{seg}$ denotes the supervised segmentation loss used in the missing-modality distillation stage and is defined as the combination of Dice loss and binary cross-entropy loss, which is used to ensure consistency between the student network’s predictions and the ground-truth segmentation labels. $L_{KD}$ represents the logit-level distillation loss, which uses mean squared error (MSE) to constrain the student network’s output ${\hat{Y}}_{S}$ to match the predicted probabilities of the teacher network ${\hat{Y}}_{T}$, with a weight coefficient $\lambda_{logit}$ set to 0.5. The teacher output is detached during optimization, and gradients are only back-propagated through the student network.

Since CMSFA is responsible for early cross-modal frequency selection, feature-level distillation is applied to its output features $F^{\left(1\right)}$. Specifically, the feature-level distillation loss $L_{FD}$ calculates the mean squared error between the CMSFA features of the full-modality teacher network and those of the student network under incomplete input conditions, with a weight coefficient of $\lambda_{feat}$ = 0.1. This constraint encourages the student network to approximate the full-modality frequency-aware representation even when one or more modalities are absent, thereby providing more stable contextual features for the subsequent decoder. From this perspective, the feature-level loss serves as an intermediate-space knowledge transfer constraint rather than merely an additional regularization term. It directly aligns the CMSFA-enhanced shallow representation of the missing-modality student with that of the full-modality teacher, which is expected to improve representation stability before progressive decoding.

During inference under missing-modality settings, only the student network is used, and unavailable modalities are masked according to the same input convention used during training. The teacher network is used only for distillation during training. Because the student is also exposed to complete inputs during distillation, its full-modality performance can be evaluated separately from the frozen teacher.

## 4. Results

### 4.1. Experimental Setup

#### 4.1.1. Datasets and Evaluation Metrics

This study conducted comprehensive experimental validation on two publicly available datasets in the field of brain tumor segmentation. The first dataset is Task01_BrainTumour from the Medical Segmentation Decathlon (MSD) [50], which contains 484 multimodal MRI cases derived from the BraTS dataset. Following the commonly used split in previous nnFormer- and SegFormer3D-based implementations [23,26], we divided the 484 labeled MSD cases into 411 training cases and 73 validation cases. The same split was used for all reproduced methods. The second dataset is BraTS 2021 [51], which also adopts the fixed partitioning scheme used in this study, with 1063 cases used for training and 188 for validation. Each case includes four MRI modalities: FLAIR, T1, T1ce, and T2. All experiments were conducted based on the aforementioned predefined partitioning to ensure fairness and reproducibility in quantitative comparisons between different models.

To avoid ambiguity regarding data independence, we further clarify that the term “case” in this manuscript refers to an anonymized 3D multimodal MRI volume with its corresponding segmentation label, rather than an individual 2D slice or a training patch. The training/validation split was performed at the case/volume level before any patch-based cropping or data augmentation. Therefore, all modalities, labels, and cropped patches derived from the same 3D MRI case were restricted to the same partition, and no case ID or 3D volume was shared between the training and validation sets. In other words, no slice-level or patch-level random splitting was performed. All experiments were conducted based on the aforementioned predefined case-level partitioning to ensure fairness and reproducibility in quantitative comparisons between different models.

It should also be noted that the reported results were obtained on predefined validation cohorts rather than on a completely independent external test cohort. This setting was adopted to maintain comparability with previous benchmark-oriented studies using the same public datasets. Therefore, the quantitative results reported in this study should be interpreted as validation-cohort performance under public benchmark settings, rather than as definitive evidence of external clinical generalization.

Evaluation metrics followed the official BraTS standard evaluation protocol, with statistics calculated separately for three tumor subregions: the whole tumor (WT), tumor core (TC), and enhancing tumor (ET) [52]. Specifically, WT is defined as the union of labels 1, 2, and 4; TC is defined as the union of labels 1 and 4; and ET corresponds to label 4. This study reports the Dice coefficient and 95% Hausdorff distance (HD95) for each tumor region. Dice score was used to quantify regional overlap between predictions and ground truth, whereas HD95 was used to evaluate boundary discrepancy while reducing the influence of extreme outliers. Other boundary-aware metrics, such as boundary intersection-over-union (boundary IoU), Surface Dice, or normalized Surface Dice, can provide more direct tolerance-based assessments of contour agreement. In this study, Dice and HD95 were selected because they are the most widely reported metrics in the BraTS evaluation protocol and in many previous brain tumor segmentation benchmarks, thereby allowing direct comparison with existing methods. In contrast, Surface Dice and boundary IoU often require additional choices of surface tolerance, boundary thickness, or implementation-specific settings, which may affect cross-study comparability. Therefore, contour-related interpretations in this study are based on HD95-supported boundary discrepancy and qualitative visual consistency, rather than a comprehensive boundary-aware metric evaluation. For case-level statistical analysis, Dice and HD95 were calculated for each validation case. The results were summarized as mean ± standard deviation, bootstrap 95% confidence interval, median, and interquartile range. The reported standard deviations were calculated across validation cases rather than across repeated training runs; therefore, they reflect inter-case heterogeneity in tumor size, morphology, boundary complexity, and subregion visibility. For HD95, undefined values may occur when the corresponding tumor subregion is absent in the prediction or ground truth, making surface-distance calculation not well defined. Therefore, the number of valid cases was explicitly reported for each HD95 metric. The detailed case-level statistical results are provided in Appendix A.

To rigorously clarify the source of baseline results, we explicitly distinguished literature-reported results from experiments reproduced and evaluated by us. For the MSD Task01_BrainTumour dataset, the results of CoTr, CoTr without CNN encoder, SETR MLA, TransUNet, UNETR, and TransBTS were taken from previously published SegFormer3D- and nnFormer-related benchmark reports. These studies used the same dataset and adopted comparable data preparation, augmentation, and WT/TC/ET reporting protocols. Since our study followed the same dataset split and largely consistent preprocessing and evaluation definitions, these results were included as literature-level benchmark references.

For the BraTS2021 dataset, we reproduced and evaluated Swin UNETR, TransBTS, nnU-Net, UNETR, and SegFormer3D using the same data split, preprocessing pipeline, training settings, and evaluation protocol as PF-CMNet. SwinBTS, 3D CATBraTS, and HDenseUNet were included as literature-reported reference results. All missing-modality experiments, including AttentionUNet3D, 3DUNet, UNETR, SegFormer3D, and PF-CMNet, were conducted by us under the same dataset split, preprocessing pipeline, modality masking strategy, segmentation label definition, and evaluation protocol.

#### 4.1.2. Implementation Details

Both training and inference for this study were performed on an NVIDIA GeForce RTX 4090 GPU (24 GB VRAM) platform, with the core algorithms implemented using the PyTorch 2.1.0 framework and the MONAI library. The datasets provide preprocessed multimodal MRI volumes, including co-registration and skull stripping. We further applied modality-wise Min-Max intensity normalization to the range [0, 1], followed by patch-based cropping during training. To balance computational efficiency and segmentation accuracy, Automatic Mixed Precision (AMP) was enabled during training. After the case-level training/validation partitioning described above, patch-based sampling was applied only within the training set. Input images from the training cases were randomly cropped into 128 × 128 × 128 3D voxel blocks, with a batch size set to 2. Validation was performed on the predefined validation cases, and no patch, slice, or volume derived from a validation case was used during model training. To enhance model generalization and prevent overfitting, online data augmentation strategies were employed, including random flipping and random rotation. To simulate real MRI truncation artifacts, Gibbs Noise and Coarse Dropout were introduced, prompting the model to learn global features rather than over-relying on local textures.

The DiceCELoss function is selected to combine the robustness of Dice Loss against class imbalance with the voxel-level discriminative power of Cross-Entropy (CE) loss. During the missing-modality distillation stage, the supervised segmentation term was implemented as Dice loss combined with binary cross-entropy, together with logit-level and feature-level distillation losses.

#### 4.1.3. Two-Stage Training Parameter Settings

To address the issue of oscillatory behavior that may arise from the direct joint optimization of the frequency-domain branch and the spatial backbone network, this study employs a two-stage training strategy based on the AdamW optimizer combined with weight decay.

The first stage serves as a stabilization and warm-up phase, during which the model is trained for 10 epochs with a learning rate of 1 × 10^−4^ to facilitate the feature fusion process within the CMSFA module. During this stage, the residual control parameter α is initialized to −2.0 and remains frozen to prevent disruptive updates from disturbing the pre-trained feature space. This initialization corresponds to an initial CMSFA residual weight of approximately 0.12 after the sigmoid transformation, which limits the early influence of the frequency-domain branch while preserving a non-zero optimization path.

In the second stage, after unfreezing all parameters, joint optimization of the entire network is performed, with fine-tuning conducted at a lower learning rate. The α parameter is released to allow the model to adaptively adjust modal weights. To prevent overfitting of the pre-trained backbone network parameters, we employ a discriminative learning rate strategy (Discriminative LR), where the learning rate for the backbone network is set to 2.5 × 10^−5^. The AdamW optimizer is used in conjunction with a polynomial learning rate scheduler (PolyLR, power = 0.9) for 150 epochs of training, while gradient clipping is introduced to enhance overall training stability. Finally, the model with the best validation set performance in the full-modality training is saved as the teacher model for the subsequent missing-modality distillation stage.

In addition to reporting the training configuration, we further provided learning-curve analysis to assess the optimization stability of PF-CMNet and the reliability of checkpoint selection. Specifically, the training/validation loss, region-wise validation Dice, region-wise validation HD95, and the corresponding learning-rate schedule were recorded and are provided in Appendix B. These curves are used as diagnostic evidence for the stability of the final full-modality optimization and model-selection process, rather than as a separate phase-by-phase visualization of every training component. The detailed analysis of these curves is presented in Section 4.5.

#### 4.1.4. Missing Modality Knowledge Distillation Protocol

To enhance the model’s robustness in scenarios with missing modalities, we introduce a two-layer teacher–student knowledge distillation framework after the full-modality training has converged. The optimal full-modality model, PF-CMNet, obtained through two-stage training, serves as the frozen static teacher network (Teacher). The student network (Student) is initialized by directly inheriting the weights of the teacher network, but during training, dynamic modality dropout is applied to its inputs, including 25% complete four-modality inputs, 50% random single-modality dropout, and 25% random dual-modality dropout. The distillation phase also employs the AdamW optimizer, with an initial learning rate set to 5 × 10^−5^, and is trained for 120 epochs using the PolyLR strategy. By simultaneously introducing constraints for both the prediction layer and feature layer distillation, the student network is guided to approximate the teacher network’s cross-modal representation capabilities, particularly maintaining consistency in shallow feature spaces. This design aims to improve segmentation stability and robustness under incomplete-input conditions by exposing the student network to different modality-availability patterns while constraining its predictions and shallow representations with the frozen full-modality teacher. For the missing-modality evaluation, the comparison models were trained under the standard full-modality setting and were directly tested with the corresponding modality masked at inference. The zero-masking protocol was used only to represent the complete absence of an input MRI sequence. It was not intended to reproduce degraded but still available MRI acquisitions, such as noise contamination, bias fields, motion artifacts, scanner-dependent intensity shifts, or local quality deterioration. These degraded-input scenarios represent a different robustness problem and were not evaluated in the present study. We did not introduce additional missing-modality training or distillation strategies into these baseline models because the purpose of this experiment was to evaluate the robustness degradation of commonly used full-modality segmentation architectures when incomplete inputs are encountered at deployment. Therefore, the experimental results presented later should be interpreted as a deployment-oriented missing-modality stress test of standard full-modality segmentation architectures, rather than a fair head-to-head comparison with dedicated incomplete-modality segmentation frameworks such as HeMIS, mmFormer, or M2FTrans.

It should be noted that the learning curves reported in Appendix B are mainly used to diagnose the stability of the full-modality optimization and checkpoint-selection process. The effectiveness of TSD-MS under incomplete-input conditions is therefore evaluated primarily through the missing-modality stress tests and ablation analysis, rather than being inferred solely from the training curves.

### 4.2. Quantitative Results

This paper systematically evaluates PF-CMNet on two public brain tumor segmentation datasets—MSD’s Task01_BrainTumour and BraTS2021—and quantitatively compares it with various representative convolutional networks, Transformers, and hybrid architecture methods. The results are shown in Table 1 and Table 2, respectively.

As shown in the table, PF-CMNet achieves stable and competitive segmentation performance on both datasets, particularly demonstrating a good balance of performance across the three tumor subregions—ET, TC, and WT. This indicates that the proposed frequency-domain-aware cross-modal modeling and progressive cross-scale detail recovery mechanism do not merely provide localized improvements in specific regions but rather exhibit good adaptability across tumor subregions of varying complexity.

On the MSD Task01_BrainTumour dataset, PF-CMNet achieved an average Dice score of 84.3%, outperforming Transformer-based segmentation methods such as UNETR, TransBTS, and TransUNet, as well as hybrid architecture methods like SegFormer3D, which achieved average Dice scores of 71.1%, 69.6%, and 64.4%, 81.5%, respectively. When examining different tumor subregions, PF-CMNet achieved Dice scores of 79.6%, 82.8%, and 90.4% for ET, TC, and WT, respectively. Notably, the ET region achieved the highest Dice score, indicating that our method possesses stronger recovery capabilities for small-volume enhancing tumor regions. Enhancing tumors typically exhibit characteristics such as small volume and irregular boundaries. The improvement in the ET region in our model is attributed to the frequency-domain-aware cross-modal interaction introduced by CMSFA in the early encoding stages, which enables more effective utilization of complementary information across different modalities. Furthermore, PF-CMNet achieves a Dice score of 90.4% in the WT region, indicating that while improving segmentation in challenging regions, the model still maintains a relatively stable overall tumor region modeling capability.

The BraTS2021 dataset provides a larger training sample size and more comprehensive data distribution coverage, resulting in an overall improvement in Dice and boundary metrics for all methods on this dataset. The advantages of PF-CMNet are also reflected in HD95-based boundary discrepancy and structural stability. Its average Dice score reached 88.2% (95% CI: 86.8–89.5%), surpassing the 87.8% of SegFormer3D and the 84.9% of TransBTS, while remaining comparable to nnU-Net and UNETR, and achieving the lowest average HD95. This indicates that our method not only maintains high region overlap accuracy but also achieves more favorable HD95-based boundary discrepancy. Further analysis of the region-specific results on BraTS2021 shows that PF-CMNet achieves Dice scores of 84.4% (95% CI: 82.3–86.2%), 88.9% (95% CI: 86.4–91.2%), and 91.4% (95% CI: 90.3–92.3%) for ET, TC, and WT, respectively. Notably, the Dice score for ET exceeds that of nnU-Net (83.1%) and HDenseUNet (83.9%), indicating that our method remains highly competitive in the challenging sub-region of tumor enhancement. Regarding the HD95 metric, PF-CMNet maintains strong competitiveness in the ET region while achieving lower HD95 values in the TC and WT regions, with the WT HD95 being the lowest result in the table. Since HD95 is more sensitive to boundary outliers and local morphological deviations, this result indicates that PF-CMNet exhibits better spatial consistency in regions with complex boundaries and irregular local structures.

A comprehensive analysis of the full-modality experimental results across the two datasets in this paper reveals that the advantages of PF-CMNet are not merely reflected in improved average Dice scores but are more evident in the restoration of challenging subregions and boundary stability. By modeling the frequency-domain complementarity between different MRI modalities during the early encoding stage, CMSFA helps enhance the discriminative information of the tumor and its core regions; the PCDF decoder improves information transfer between shallow spatial details and deep semantic features through progressive cross-scale detail fusion, thereby enhancing the structural integrity of the prediction results.

The results of the deployment-oriented missing-modality stress test are shown in Table 3. Here, “w/o” denotes “without,” meaning that the corresponding MRI modality was set to zero during inference. For baseline models, this masking was applied only at inference after standard full-modality training, whereas PF-CMNet used the distilled student trained with TSD-MS. The full-modality baseline models showed varying degrees of performance degradation when tested with masked modalities, indicating different sensitivities to incomplete inputs. In contrast, the PF-CMNet student trained with TSD-MS showed improved robustness under the same modality-masked inference settings, suggesting that the proposed distillation strategy can mitigate the representation degradation observed in standard full-modality models. AttentionUNet3D exhibits significant performance degradation under various missing modality scenarios; for example, under w/o FLAIR and w/o T1 + T2 conditions, its average Dice scores are only 5.52% and 8.39%, respectively, indicating that this model is highly sensitive to critical input modalities. 3DUNet performs relatively stably under certain single-modality missing conditions, but in the “no T1ce” scenario, the average Dice score drops to 42.56%, with the ET Dice score falling to just 3.75%, indicating that traditional convolutional architectures struggle to maintain stable, fine-grained segmentation when information essential for tumor discrimination is missing. UNETR and SegFormer3D maintain a certain level of whole-tumor segmentation capability in some scenarios but still exhibit significant fluctuations under w/o FLAIR or dual-modality missing conditions, reflecting that relying solely on global modeling or hierarchical Transformer encoding cannot fully address the representation degradation caused by missing modalities.

Under this deployment-oriented stress-test setting, the PF-CMNet student trained with TSD-MS showed smaller performance degradation and higher average Dice scores than the evaluated standard full-modality baselines under modality-masked inference. Under conditions without FLAIR, T1, T1ce, T2, and T1 + T2, PF-CMNet achieved average Dice scores of 78.64%, 82.58%, 58.39%, 82.03%, and 79.29%, respectively, which were higher than those of the evaluated full-modality baselines under modality-masked inference. Particularly in the challenging scenarios of w/o FLAIR and w/o T1 + T2, the distilled PF-CMNet student achieved mean Dice improvements of 23.14 and 22.78 percentage points over the strongest evaluated full-modality baseline under modality-masked inference, indicating greater stability under conditions of critical modality loss and dual-modality loss.

Across different tumor subregions, the PF-CMNet student showed relatively stable performance on WT and TC under the evaluated modality-masked inference settings. For WT, our method achieved Dice scores of 81.31%, 90.33%, 90.04%, 89.22%, and 88.13% under the five missing data scenarios, respectively, showing more stable whole-tumor region reconstruction than the evaluated standard full-modality baselines under modality-masked inference. For TC, the PF-CMNet student achieved Dice scores of 78.82% and 76.59% under the challenging w/o FLAIR and w/o T1 + T2 conditions, respectively, indicating more stable tumor-core localization than the evaluated standard full-modality baselines under modality-masked inference.

For the ET region, all methods showed a significant decline under the w/o T1ce condition, as the discrimination of the enhancing tumor relies heavily on the contrast-enhanced information provided by T1ce. Nevertheless, PF-CMNet still achieved the highest ET Dice score in this scenario, reaching 22.13%, outperforming UNETR (12.99%), SegFormer3D (5.48%), and 3DUNet (3.75%). Additionally, under w/o FLAIR, w/o T1, w/o T2, and w/o T1 + T2 conditions, the ET Dice values of our method reached 75.79%, 76.31%, 76.38%, and 73.15%, respectively, suggesting that TSD-MS helps preserve ET-related discriminative responses when standard full-modality baselines degrade under modality-masked inference.

In summary, PF-CMNet not only exhibits strong stability under single-modality missing conditions but also maintains high segmentation performance in the more challenging scenario of dual-modality missing data. This indicates that frequency-domain-aware cross-modal modeling enhances the utilization of complementary information between the remaining modalities, the detail-recovery decoding architecture helps improve the spatial continuity of tumor regions, and the teacher–student-based missing-modality distillation further mitigates the degradation of discriminative representations caused by incomplete inputs. Overall, the results in Table 3 support the effectiveness of TSD-MS in improving PF-CMNet’s robustness under the evaluated missing-modality stress-test settings.

### 4.3. Qualitative Results

**Figure 5:**
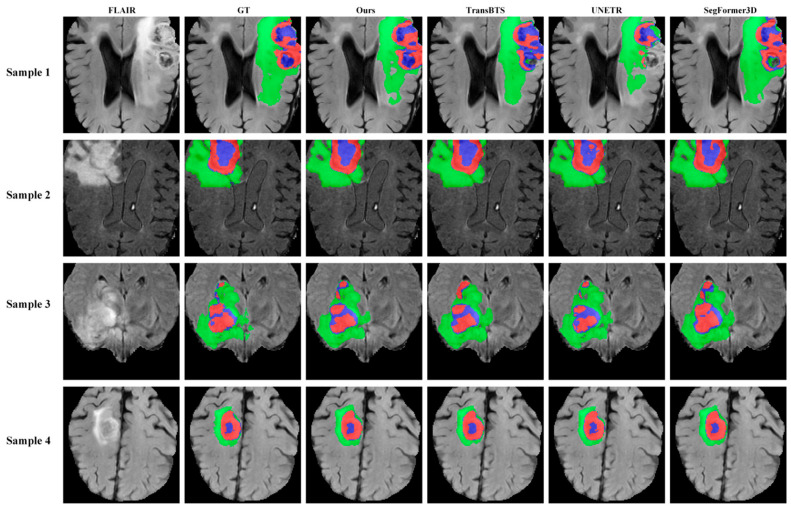
Qualitative comparison of segmentation results under full-modality input. Representative axial slices are shown with FLAIR as the reference image. Green indicates peritumoral edema, red indicates non-enhancing tumor core, and blue indicates enhancing tumor.

To provide a more intuitive analysis of the segmentation performance of different methods, Figure 5 presents the visualization results of PF-CMNet and several representative methods on typical cases. Four representative samples were selected from the validation set for qualitative demonstration, including cases with relatively clear tumor boundaries and challenging cases with small enhancing tumor regions, blurred tumor-core transitions, or irregular lesion morphology. All results are visualized in the axial plane, with different colors corresponding to different tumor subregions, and the FLAIR sequence serving as the primary reference modality. Overall, in cases with larger tumor regions and relatively clear boundaries, most methods can achieve reasonably accurate segmentation results; however, in challenging cases where the enhancing tumor region is small, the transition between the tumor core and surrounding edema is blurred, or the boundaries exhibit significant irregular deformation, the differences among the various methods become more pronounced. Compared to SegFormer3D and other representative Transformer-based methods, PF-CMNet typically restores more complete local ET structures and maintains a more continuous distribution of WT regions in the selected difficult cases, indicating that the proposed method exhibits better visual consistency in handling small-volume lesions and preserving complex spatial structures.

Further observation reveals that the compared methods are more prone to two typical errors. The first is the failure to segment small-volume enhancing tumor, which is typically associated with insufficient utilization of shallow-layer discriminative information; the second is excessive smoothing of complex boundaries and transition regions, reflecting limited cross-scale detail integration capabilities during the decoding stage. The visual improvements achieved by our method in these challenging cases indicate that the frequency-domain-aware cross-modal enhancement introduced at the encoding stage helps extract more discriminative complementary information at an early stage, while the progressive cross-scale detail fusion decoder further transforms these shallow-level enhanced features into more complete regional reconstruction results and more continuous spatial structures. These visual improvements are primarily manifested in the restoration of small lesions, regional integrity, and local structural continuity, consistent with the trend of improved Dice scores observed in the quantitative results discussed earlier.

**Figure 6:**
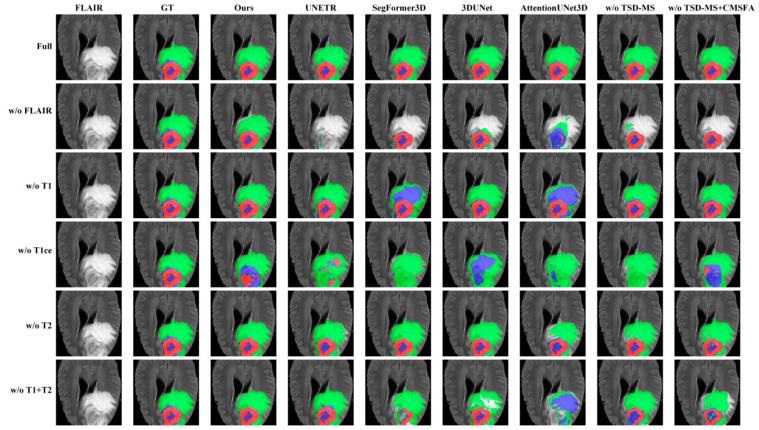
Qualitative comparison under missing-modality settings. Rows correspond to Full, w/o FLAIR, w/o T1, w/o T1ce, w/o T2, and w/o T1 + T2 inputs, where “w/o” denotes the absence of the corresponding modality. Columns show the FLAIR image, ground truth, and predictions of different methods. Green indicates peritumoral edema, red indicates non-enhancing tumor core, and blue indicates enhancing tumor.

Furthermore, to further validate the stability and cross-modal robustness of our method under incomplete input conditions, Figure 6 presents visualization results for typical cases in our dataset under different missing-modality settings. From top to bottom, the figure corresponds to complete input (Full), single-modality missing (w/o FLAIR, w/o T1, w/o T1ce, w/o T2), and dual-modality missing (w/o T1 + T2) scenarios; from left to right, it shows the FLAIR image, the ground truth, and the prediction results of different methods. Different colors represent different tumor subregions, allowing for the observation of each method’s regional recovery and structural preservation capabilities as modal information is progressively reduced.

This paper selects four representative 3D medical image segmentation models as comparison baselines, including 3D U-Net, Attention U-Net3D, UNETR, and SegFormer3D. These models represent different mainstream architectural paradigms in the current field of volumetric segmentation: 3D U-Net represents the classic convolutional encoder–decoder architecture; Attention U-Net3D represents a convolutional-enhanced architecture incorporating local attention mechanisms; UNETR represents a pure Transformer-based volumetric modeling method; and SegFormer3D represents a lightweight hierarchical Transformer encoding combined with an efficient multi-scale decoding architecture. By covering three typical approaches—CNNs, attention-enhanced CNNs, and Transformers—we can more comprehensively analyze the differences in robustness among various modeling mechanisms under missing-modality conditions. Since missing-modality experiments require all compared methods to be retrained and evaluated under a unified data split, identical training strategies, and consistent missing-modality protocols, this study prioritizes representative models with distinct architectural differences for comparison to ensure the fairness and interpretability of the experimental results.

The visualization results show that, under complete input conditions, most methods can effectively recover the primary tumor region. However, when key modalities such as FLAIR or T1ce images are missing, performance disparities among different methods become significantly more pronounced. Since the enhancement patterns of tumor regions are highly sensitive to the discriminative information provided by T1ce images, some contrast-based methods exhibit obvious issues in scenarios where T1ce images are missing, such as failure to segment enhancing tumor. Under conditions where two modalities are missing, the WT regions of certain methods exhibit boundary contraction or localized expansion, reflecting these methods’ dependence on the completeness of modal inputs. In contrast, under the evaluated modality-masked inference settings, the PF-CMNet student maintained relatively continuous WT regions and relatively stable TC spatial positions in most displayed cases, preserving an overall tumor morphology closer to the reference label.

Since the enhancement of tumor regions relies heavily on the contrast-enhanced information provided by T1ce, the difficulty of reconstructing ET under w/o T1ce conditions increases significantly. This physiological feature, generated by contrast agent uptake, cannot be fully derived from FLAIR, T1, or T2. Therefore, all methods exhibit varying degrees of ET degradation in this scenario. Nevertheless, PF-CMNet still retains more reasonable core region contours and local ET responses compared to other methods, indicating that the proposed cross-modal enhancement mechanism can more fully utilize complementary discriminative cues from the remaining modalities.

**Figure 7:**
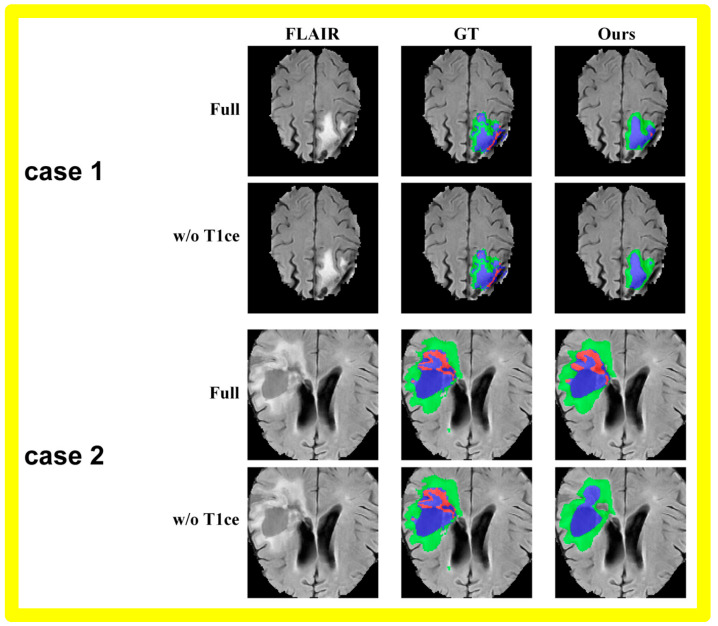
Representative failure cases of PF-CMNet. Case 1 shows missed or incomplete ET segmentation under the w/o T1ce setting. Case 2 shows local boundary instability in regions with blurred tumor-core transitions and irregular edema morphology. These examples illustrate the remaining limitations of PF-CMNet under low-contrast or highly ambiguous anatomical conditions. Green indicates peritumoral edema, red indicates non-enhancing tumor core, and blue indicates enhancing tumor.

To avoid over-interpreting qualitative results from only visually favorable examples, we further provide representative failure cases of PF-CMNet in Figure 7. The first typical failure pattern occurs when the T1ce modality is unavailable. Because enhancing tumor delineation strongly depends on contrast-enhanced information, PF-CMNet may still miss small or weakly enhanced ET regions under the w/o T1ce setting, even though the distilled student model is more stable than the evaluated full-modality baselines. The second failure pattern appears in cases with blurred tumor-core boundaries or highly irregular edema–tumor transitions, where the model may produce locally discontinuous predictions or slight over-segmentation around ambiguous boundary regions. These examples indicate that PF-CMNet does not fully solve the segmentation of low-contrast enhancing tumor regions or anatomically ambiguous tumor boundaries, and they are consistent with the quantitative observation that ET segmentation remains the most sensitive to missing T1ce information.

### 4.4. Model Efficiency Analysis

In practical clinical applications, the memory footprint and computational complexity of segmentation models are as important as their segmentation accuracy. To validate the practical inference efficiency of the proposed model, we compared PF-CMNet with several representative methods, including UNETR, TransBTS, nnU-Net, and Swin UNETR, on the BraTS2021 dataset. The comparison metrics included the number of model parameters (Params), floating-point operations (FLOPs), peak inference memory consumption, and average Dice score. The detailed results are presented in Table 4.

The results demonstrate that the proposed PF-CMNet achieves a favorable balance between segmentation performance and computational cost. While maintaining a competitive average Dice score of 88.2%, PF-CMNet contains only 27.88 M parameters. In contrast, models with segmentation performance comparable to PF-CMNet, such as nnU-Net and Swin UNETR, require substantially larger parameter sizes and computational overhead. Specifically, Swin UNETR consumes up to 739.52 G FLOPs and 3.94 GB of peak inference memory, with memory consumption approximately seven times higher than that of the proposed method. Compared with Swin UNETR, PF-CMNet reduces the number of parameters and computational cost by approximately 55.17% and 54.52%, respectively. Compared with nnU-Net, the reductions are 10.64% and 30.33%, respectively.

These results indicate that PF-CMNet provides a more favorable accuracy–memory trade-off than several representative high-performing 3D segmentation models under the same benchmarking setting. By maintaining moderate memory consumption and computational burden, the proposed method still achieves strong segmentation performance.

### 4.5. Training-Dynamics Analysis

To provide empirical evidence for the optimization stability of PF-CMNet, we further analyzed the training dynamics of the main full-modality training process, including the training/validation loss curves, region-wise validation Dice curves, validation HD95 curves, and the learning-rate schedule, as shown in Appendix B.

**Figure A1:**
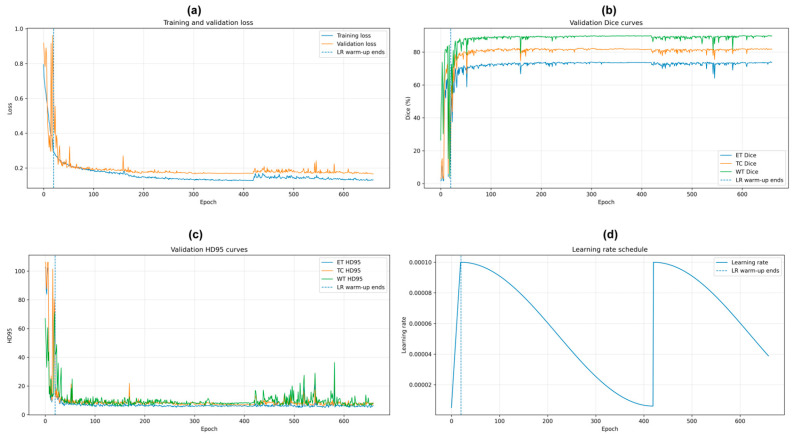
Training-dynamics curves of PF-CMNet during the main full-modality training process. (**a**) Training and validation loss curves. (**b**) Region-wise validation Dice curves for ET, TC, and WT. (**c**) Region-wise validation HD95 curves for ET, TC, and WT. (**d**) Learning-rate schedule with warm-up and cosine annealing.

As shown in Figure A1a, the training loss decreased rapidly during the early optimization stage and then gradually entered a low and stable range. The validation loss showed relatively large fluctuations in the initial epochs, which is expected because the model had not yet established stable multimodal feature representations at the beginning of training. After the early stage, the validation loss decreased substantially and remained within a relatively stable range. Although several local oscillations appeared in the later epochs, these fluctuations were not accompanied by a continuously increasing validation loss or a progressively widening gap between training and validation loss. Therefore, the loss curves do not indicate severe or sustained overfitting during the main full-modality training process.

Figure A1b presents the region-wise validation Dice curves for ET, TC, and WT. The Dice scores of all three tumor subregions increased sharply during the early epochs and then gradually reached stable plateaus. WT achieved the most stable and highest Dice values, which is consistent with the relatively larger spatial extent of the whole tumor region. TC also showed a stable convergence trend after the early optimization stage. In comparison, ET exhibited more visible fluctuations, which can be attributed to the smaller volume, irregular morphology, and higher boundary sensitivity of enhancing tumor regions. Nevertheless, the ET Dice curve did not show persistent degradation in the later stage, suggesting that the model maintained stable segmentation ability for small and challenging tumor subregions after convergence.

Figure A1c shows the validation HD95 curves for the three tumor subregions. In the early epochs, HD95 values were high and unstable, reflecting inaccurate boundary localization before the model had sufficiently learned tumor morphology and spatial structure. As training progressed, the HD95 values decreased markedly and then remained within a lower range after convergence, indicating improved boundary agreement between predictions and ground-truth labels. Occasional HD95 spikes were still observed in the later epochs, especially for boundary-sensitive regions. This phenomenon is reasonable because HD95 is more sensitive than Dice to a small number of difficult validation cases, small-region prediction errors, and local boundary deviations. Importantly, these spikes were transient and did not form a sustained upward trend, suggesting that the overall boundary localization performance remained stable.

Figure A1d illustrates the learning-rate schedule used during training. The model adopted a warm-up strategy combined with cosine annealing. During the warm-up stage, the learning rate gradually increased to stabilize early optimization, especially considering that PF-CMNet introduces the CMSFA frequency-aware branch and requires stable alignment between the frequency-enhanced representation and the baseline spatial feature space. After warm-up, the learning rate followed a cosine annealing schedule and gradually decreased, allowing the model to enter a more stable convergence phase. Around the learning-rate transition or restart-like adjustment point, short-term oscillations can be observed in the validation loss and boundary-sensitive metrics. However, these oscillations were consistent with the learning-rate schedule and were not associated with continuous deterioration in validation Dice or HD95.

Taken together, the four learning-curve plots indicate that PF-CMNet underwent a typical and healthy optimization process: the training loss decreased effectively, the validation loss did not show a sustained overfitting pattern, the validation Dice curves reached stable plateaus, and the validation HD95 curves decreased and remained relatively stable after convergence. The final checkpoint was selected using early stopping after the validation performance had entered a stable plateau, rather than relying on a single accidental validation peak. Therefore, the reported full-modality validation performance is supported by stable training dynamics and is unlikely to be the result of an isolated fluctuation during optimization.

### 4.6. Ablation Analysis

To validate the effectiveness of the method proposed in this paper, we conducted ablation experiments on the MSD Task01_BrainTumour dataset, with the results shown in Table 5. Recent hybrid AI–Taguchi–ANOVA studies have highlighted the value of structured experimental optimization and factor-contribution analysis for interpreting model behavior [61]. Inspired by this methodological perspective, the present ablation study analyzes the contribution of PCDF, CMSFA, and TSD-MS to full-modality segmentation performance and missing-modality robustness. Here, B denotes the baseline segmentation architecture consisting of a hierarchical Transformer encoder and an MLP decoder.

From the multimodal results, it is evident that the gradual introduction of structural modules consistently improves the model’s baseline segmentation performance. The Full Mean Dice score of the baseline architecture B is 81.48%; replacing the original MLP decoder with PCDF improves it to 82.15%, and further introducing CMSFA boosts it to 84.25%. This demonstrates the advantages of PCDF and CMSFA in cross-scale recovery capabilities and cross-modal complementary information modeling, thereby enhancing overall segmentation performance under complete input conditions.

However, under missing-modality conditions, the training objective of TSD-MS is primarily focused on missing-modality scenarios. At this stage, the full-modality PF-CMNet serves as a fixed teacher network providing stable supervision, while the student network learns through random missing-modality inputs to approximate the teacher’s discriminative response under full-input conditions. Consequently, the performance ceiling under full-input conditions is essentially determined by the teacher model’s B + PCDF + CMSFA architecture; therefore, results for full-input conditions are not reported separately.

Following the introduction of TSD-MS, the distilled student model achieved significantly higher Dice scores across all evaluated missing-modality stress test scenarios. Under conditions of missing FLAIR, missing T1, missing T1ce, missing T2, and missing T1 + T2 dual-modality data, the complete model achieved average Dice scores of 78.64%, 82.58%, 58.39%, 82.03%, and 79.29%, respectively. Notably, under the extreme condition of missing T1 + T2 dual-modality data, the complete model still maintained an average Dice score of 79.29%, whereas the B, B + PCDF, and B + PCDF + CMSFA baseline models achieved only 14.45%, 22.72%, and 25.35%. These results indicate that the overall TSD-MS strategy, which combines random modality dropout with teacher-guided logit-level and feature-level supervision, plays an important role in mitigating performance degradation under incomplete input conditions. Since TSD-MS jointly involves incomplete-input exposure and teacher-guided supervision, the present ablation mainly verifies the effectiveness of the complete TSD-MS strategy. A more fine-grained statistical decomposition of the separate contributions of modality dropout, logit distillation, and feature distillation will be explored in future work using more structured experimental optimization methods.

The ablation results in the table indicate that PCDF and CMSFA primarily enhance the multimodal segmentation backbone network, while TSD-MS is the key factor in improving robustness under the evaluated missing-modality scenarios. Furthermore, the missing-modality ablation experiments in this paper serve as a stress test analysis rather than a fully controlled comparison with dedicated missing-modality learning methods. Additionally, the current evaluation reports only four single-modality missing-modality settings and one dual-modality missing-modality setting and does not cover all possible non-empty modality combinations. Therefore, more comprehensive missing-modality evaluations are still needed in the future, incorporating additional baseline models trained using the same modality dropout protocol.

## 5. Discussion

The above results still require a multifactorial interpretation. First, the missing-modality experiments in this paper were designed as deployment-oriented stress tests. These experiments target complete input-sequence absence rather than all clinically degraded acquisition scenarios. Zero masking of an unavailable MRI modality represents a sequence that is not acquired, corrupted beyond use, or unavailable to the segmentation model. However, this protocol does not reproduce partially degraded but still informative images affected by noise, bias fields, motion artifacts, scanner-dependent intensity shifts, or local quality deterioration. Therefore, the missing-modality results should be interpreted as evidence of robustness to complete modality absence, rather than as evidence of robustness to all non-ideal MRI acquisition conditions. The comparison models were all trained under standard full-modality settings, with the corresponding modalities masked directly during the inference stage; in contrast, PF-CMNet was evaluated using a distilled student model trained via TSD-MS. The results for missing modalities primarily reflect the degradation in robustness of standard full-modality models under missing-modality inference, as well as the mitigating effect of the proposed distillation strategy on this degradation. This mitigating effect should be understood as the combined result of incomplete-input exposure and teacher-guided knowledge transfer. Random modality dropout exposes the student network to variable input availability, whereas logit-level distillation constrains the final predictive distribution and CMSFA feature-level distillation constrains the intermediate shallow representation. This dual-level supervision provides a more stable learning target than supervised segmentation loss alone under incomplete inputs. Nevertheless, we acknowledge that the current study does not provide a formal statistical decomposition of the separate contributions of modality dropout, logit distillation, and feature distillation. Further controlled analyses of convergence behavior, feature-space alignment, and component-level contribution will be valuable for more comprehensively interpreting the knowledge transfer mechanism in TSD-MS. A rigorous and fair comparison with dedicated incomplete-modality segmentation frameworks, such as HeMIS, mmFormer, M2FTrans, and recent modality-completion or modality-robust fusion methods, has not yet been conducted. These methods are specifically trained for incomplete multimodal inputs and should be compared under unified data splits, preprocessing pipelines, missing-modality sampling protocols, and training budgets. In this study, we did not include them because our missing-modality experiment was designed as a deployment-oriented stress test of standard full-modality models rather than a comprehensive benchmark of specialized incomplete-modality segmentation frameworks. Future work will incorporate these dedicated methods under the same modality-dropout training and evaluation protocol.

In addition, the current missing-modality evaluation reports on four single-modality missing-modality settings and one dual-modality missing-modality setting. Although these settings have clinical relevance and can reveal the sensitivity of different models to incomplete inputs, they do not cover all 15 non-empty modality combinations formed by the four MRI sequences. A more comprehensive evaluation, including additional dual-modality missing-modality and single-modality available scenarios, will be conducted in subsequent experiments.

The w/o T1ce setting remains highly challenging, particularly in the segmentation of enhancing tumor regions. This is consistent with the high dependence of enhancing tumor contouring on T1-weighted information. Although the distilled PF-CMNet student model retains more local ET responses under modal masking inference compared to the evaluated full-modal baseline models, the ET Dice score still drops significantly when T1ce is missing. This indicates that the missing contrast-enhanced information cannot be fully inferred from the remaining modalities; future work will further explore uncertainty-aware prediction, modal prior modeling, or modal completion strategies tailored to this scenario.

Although CMSFA introduces an explicit frequency-aware design, we acknowledge that the current low-, mid-, and high-frequency division is a structured modelling choice rather than a rigorously derived physical model of MRI tissue contrast. Multimodal MRI brain tumor segmentation is affected by complex factors, including tissue relaxation properties, acquisition protocols, contrast enhancement, tumor heterogeneity, and image reconstruction effects. The current study uses frequency-domain decomposition as an engineering representation to enhance cross-modal feature modelling. More rigorous physics-informed validation, spectral statistical analysis, and band-wise sensitivity evaluation will be investigated in future work.

The fixed number of frequency bands is another methodological constraint of the present implementation. In CMSFA, the number of frequency bands was set to K = 3 to provide a compact low-/mid-/high-frequency decomposition and to balance interpretability with computational cost. However, we did not perform an exhaustive sensitivity analysis over different K values. Therefore, K = 3 should be interpreted as a conservative design choice rather than a theoretically optimal setting. Future work will systematically investigate the influence of different frequency-band numbers on segmentation accuracy, HD95-based boundary discrepancy, missing-modality robustness, and computational complexity.

The boundary-related analysis in this study also remains limited. Although HD95 is a standard surface-distance metric in BraTS-style evaluation, it does not fully characterize local contour overlap or tolerance-based boundary agreement. In addition, the qualitative visualizations provide intuitive examples of tumor contour recovery but cannot replace a comprehensive boundary-aware metric evaluation. Future studies will include additional boundary-aware metrics, such as Surface Dice, normalized Surface Dice, boundary IoU, and average symmetric surface distance, to provide a more complete assessment of tumor contour accuracy.

Finally, dedicated interpretability analyses were not included in the current study. Although the proposed CMSFA module explicitly introduces frequency-band statistics and spatial gating to guide cross-modal feature modulation, its effectiveness was mainly evaluated through segmentation performance, ablation experiments, and qualitative segmentation results. Therefore, the current manuscript does not provide direct visual evidence, such as attention maps, Grad-CAM, or frequency-response visualizations, showing that the learned responses are spatially concentrated on specific anatomical tumor regions. Future work will incorporate systematic interpretability analyses, including attention visualization, frequency-response mapping, and Grad-CAM-based feature attribution, to better understand how PF-CMNet exploits tumor-related anatomical and cross-modal information.

PF-CMNet was evaluated on two public brain tumor segmentation datasets, namely MSD Task01_BrainTumour and BraTS2021, using predefined case-level training/validation partitions. In these experiments, no case ID or 3D volume was shared between the training and validation sets, and all patch-based sampling was performed only after case-level partitioning. This setting avoids slice-level or patch-level leakage from the same volume and follows the benchmark-oriented evaluation setting commonly adopted in previous nnFormer- and SegFormer3D-based implementations. However, the present study did not include a completely independent external held-out test cohort with scanner-specific metadata, nor did it perform scanner-stratified validation across different vendors, field strengths, or institutional imaging protocols. In addition, the predefined validation cohort was used for checkpoint selection and final quantitative reporting, which may introduce optimistic bias compared with evaluation on a completely untouched external test set. Therefore, the reported results should be interpreted as performance on predefined public validation cohorts rather than as definitive evidence of external clinical generalization. Future work will further evaluate PF-CMNet on independent multicenter cohorts, scanner-stratified datasets, and prospective clinical workflow data to more rigorously assess its generalization capability across acquisition protocols, scanning devices, and real-world data distributions. External validation on independent multicenter clinical data is still required to assess the model’s generalization capabilities across different acquisition protocols, scanning devices, and data distributions. Although the motivation for this study relates to downstream image-guided neurosurgical planning and 3D visualization, this paper does not include mixed-reality navigation experiments, registration accuracy evaluations, or clinical user studies; therefore, the clinical deployment value of PF-CMNet requires further validation.

From a clinical workflow perspective, PF-CMNet is intended to serve as an upstream segmentation component rather than an algorithm running directly on a mixed-reality headset or intraoperative display device. A practical deployment pipeline may include multimodal MRI acquisition, image preprocessing, PF-CMNet-based tumor segmentation on a workstation or server-side GPU, clinician review and correction, 3D reconstruction of the tumor and surrounding anatomical structures, registration to the patient space, and subsequent visualization in a surgical navigation or mixed-reality system. Under this workflow, the computationally intensive segmentation step can be completed before surgery or during preoperative planning, while the intraoperative platform mainly receives the reconstructed anatomical models for visualization and navigation. Therefore, the relatively low inference memory consumption of PF-CMNet may facilitate integration into workstation-based neurosurgical planning systems. However, real-time intraoperative updating, registration accuracy, brain-shift compensation, and user interaction still require dedicated system-level validation.

From the perspective of clinical transferability and the use of the model in real-world clinical settings, the current validation results should be regarded as proof of concept rather than direct validation of clinical readiness. Although PF-CMNet achieved competitive Dice and HD95 scores on public benchmark datasets and demonstrated greater robustness in predefined missing-modality stress tests, these evaluations do not fully reflect the complexity of real-world clinical scenarios. In practical applications, segmentation performance may be compromised by factors such as severe motion artifacts, scanner-specific intensity shifts, suboptimal image quality control, atypical tumor morphology, postoperative anatomical changes, and variations in preprocessing workflows. Therefore, before using PF-CMNet’s segmentation results for surgical planning or image-guided navigation, its clinical application should require standardized preprocessing, uncertainty estimation, automatic fault detection, and closed-loop validation involving clinicians. Future work will further evaluate model calibration, robustness to acquisition artifacts, external multicenter generalization, and prospective clinical applicability to determine whether PF-CMNet can provide reliable support within real-world neurosurgical workflows.

The efficiency benchmark further suggests that PF-CMNet achieves a favorable accuracy–memory trade-off among the compared models. However, the reported parameter count, FLOPs, and inference memory consumption were measured on a single RTX 4090 GPU under a fixed inference configuration. These values may vary with hardware platforms, software environments, input volume sizes, and sliding-window inference settings. In addition, the current efficiency analysis focuses on model-level computational cost and inference memory consumption, and does not include the complete clinical workflow, such as image transfer, preprocessing, registration, 3D reconstruction, or mixed-reality visualization. Future work will further evaluate the end-to-end runtime performance of PF-CMNet in practical image-guided neurosurgical workflows.

In future work, we will further validate the framework’s adaptability to various lesion morphologies, imaging distributions, and task scenarios, thereby advancing this framework toward a more generalizable and robust medical image segmentation method.

## 6. Conclusions

This study proposes PF-CMNet, a Progressive Frequency-Aware Cross-Modal Network with Missing-Modality Distillation, for 3D multimodal brain tumor segmentation. Specifically, we introduce CMSFA in the early stages of the encoder to explicitly enhance the complementary representations across different MRI modalities, while combining it with PCDF to effectively improve the model’s ability to recover complex boundaries and small-volume lesions. Finally, by designing a teacher–student two-stage knowledge distillation mechanism, we transfer the discriminative knowledge learned under full-modality conditions to missing-modality scenarios. Through joint constraints on the feature and prediction layers during training, we enhance the model’s segmentation stability and robustness under missing-modality input conditions. Experimental results on the public MSD brain tumor segmentation task and the BraTS2021 dataset demonstrate that our proposed PF-CMNet achieves competitive performance on both datasets and exhibits greater robustness under missing-modality conditions.

These findings indicate that the proposed framework achieves a favorable balance among segmentation accuracy, boundary detail recovery, missing-modality robustness, and model-level computational practicality. Nevertheless, further validation on independent multicenter clinical data and workflow-level neurosurgical systems is still required before clinical deployment.

## Figures and Tables

**Table 1:** Quantitative analysis on the MSD Task01_BrainTumour dataset. Dice coefficients and HD 95% of ET, TC, and WT were used as evaluation metrics. The up arrow (↑) indicates that higher values are better, while the down arrow (↓) indicates that lower values are better.

Method	Dice Score (%) ↑				Hausdorff 95% (mm) ↓			
	ET	TC	WT	Avg.	ET	TC	WT	Avg.
CoTr [53]	55.7	74.8	74.6	68.3	9.45	10.45	9.20	9.70
CoTr w/o CNN Encoder [53]	52.3	69.8	71.2	64.4	9.59	12.58	11.49	11.22
SETR MLA [54]	55.4	66.5	69.8	63.9	10.24	14.72	15.50	13.49
UNETR [21]	58.5	76.1	78.9	71.1	9.35	8.85	8.27	8.82
TransBTS [37]	57.4	73.5	77.9	69.6	9.97	8.95	10.03	9.65
TransUNet [55]	54.2	68.4	70.6	64.4	10.42	14.5	14.03	12.98
SegFormer3D [26]	73.0	81.9	89.6	81.5	6.08	6.88	7.15	6.71
Ours	79.6	82.8	90.4	84.3	6.00	7.40	7.49	6.96

**Table 2:** Quantitative analysis of the BraTS2021 dataset. Dice coefficients and HD 95% of ET, TC, and WT were used as evaluation metrics. The up arrow (↑) indicates that higher values are better, while the down arrow (↓) indicates that lower values are better.

Method	Dice Score (%) ↑				Hausdorff 95% (mm) ↓			
	ET	TC	WT	Avg.	ET	TC	WT	Avg.
SwinBTS [56]	83.2	84.8	91.8	86.6	16.03	14.51	3.65	11.39
3D CATBraTS [57]	79.2	78.4	85.1	80.9	6.92	9.47	13.71	10.03
HDenseUNet [58]	83.9	89.7	91.4	88.3	3.67	4.56	5.23	4.49
Swin UNETR [25]	85.3	87.6	92.7	88.5	3.21	4.13	4.76	4.03
TransBTS [37]	81.8	85.0	88.0	84.9	16.79	11.14	12.78	13.57
nnU-Net [59]	83.1	89.3	90.9	87.8	3.89	4.78	5.34	4.67
UNETR [21]	85.2	86.6	92.2	88.0	12.26	7.73	7.78	9.26
SegFormer3D [26]	83.5	89.4	90.4	87.8	4.79	4.80	6.17	5.25
Ours	84.4	88.9	91.4	88.2	4.09	3.93	3.74	3.92

**Table 3:** Deployment-oriented missing-modality stress test on the MSD Task01_BrainTumour dataset. The up arrow (↑) indicates that higher values are better.

Type	Method	Dice Score (%) ↑				
		w/o FLAIR	w/o T1	w/o T1ce	w/o T2	w/o T1 + T2
**WT**	AttentionUNet3D [60]	7.54	67.73	73.71	38.79	17.93
	3DUNet [18]	48.24	85.18	83.63	80.72	61.17
	UNETR [21]	6.09	65.74	71.60	71.68	57.69
	SegFormer3D [26]	29.38	81.95	78.43	79.99	30.13
	Ours	81.31	90.33	90.04	89.22	88.13
**TC**	AttentionUNet3D	5.82	56.96	25.01	33.90	6.59
	3DUNet	62.68	78.06	40.30	67.14	55.56
	UNETR	9.73	52.23	37.44	46.06	35.35
	SegFormer3D	48.23	62.17	8.00	50.61	4.38
	Ours	78.82	81.12	63.00	80.50	76.59
**ET**	AttentionUNet3D	3.20	52.41	5.48	19.51	0.65
	3DUNet	55.57	75.50	3.75	67.31	52.81
	UNETR	10.41	51.51	12.99	41.86	37.35
	SegFormer3D	44.27	64.13	5.48	56.75	8.84
	Ours	75.79	76.31	22.13	76.38	73.15
**Mean**	AttentionUNet3D	5.52	59.03	34.73	30.73	8.39
	3DUNet	55.50	79.58	42.56	71.72	56.51
	UNETR	8.74	56.49	40.68	53.20	43.46
	SegFormer3D	40.63	69.42	30.64	62.45	14.45
	Ours	78.64	82.58	58.39	82.03	79.29

**Table 4:** Efficiency analysis on the BraTS 2021 dataset. This table compares the parameter count (Params), floating-point operations (FLOPs), peak inference memory (GB), extra inference memory (GB), and average Dice score. All benchmarking analyses were conducted on a single NVIDIA RTX 4090 GPU. The up arrow (↑) indicates that higher values are better, while the down arrow (↓) indicates that lower values are better.

Method	Params (M) ↓	FLOPs (G) ↓	Peak Inf. Mem. (GB) ↓	Extra Inf. Mem. (GB) ↓	Avg. Dice (%) ↑
UNETR	102.45	178.81	1.43	0.98	88.0
TransBTS	32.99	326.24	1.44	1.25	84.9
nnU-Net	31.20	482.80	2.33	2.15	87.8
Swin UNETR	62.19	739.52	3.94	3.64	88.5
Ours	27.88	336.35	0.56	0.40	88.2

**Table 5:** Ablation study of the proposed modules under full-modality and missing-modality settings. The up arrow (↑) indicates that higher values are better.

Method	Mean Dice Score(%) ↑					
	Full	w/o FLAIR	w/o T1	w/o T1ce	w/o T2	w/o T1 + T2
B	81.48	40.63	69.42	30.64	62.45	14.45
B + PCDF	82.15	31.01	61.18	36.04	61.10	22.72
B + PCDF + CMSFA	84.25	34.77	61.42	34.49	67.40	25.35
B + PCDF + CMSFA + TSD-MS	—	78.64	82.58	58.39	82.03	79.29

## Data Availability

The datasets used in this study are publicly available. The MSD Task01_BrainTumour dataset is available from the Medical Segmentation Decathlon repository at http://medicaldecathlon.com/. (accessed on 1 May 2026) The BraTS2021 dataset is available from the RSNA-ASNR-MICCAI Brain Tumor Segmentation Challenge repository, subject to the corresponding data access policies, at https://www.synapse.org/#!Synapse:syn25829067/wiki/610863 (accessed on 1 May 2026).

## Appendix A

Note: Standard deviations were calculated from case-level validation results rather than repeated training runs. Since Dice and HD95 distributions can be skewed, median and interquartile range are also reported.

**Table A1:** Case-level statistical results of PF-CMNet on the BraTS2021 validation set.

Metric	Mean ± SD	95% CI	Median	IQR
Dice ET (%)	84.39 ± 13.84	[82.26, 86.22]	88.83	81.63–91.77
Dice TC (%)	88.93 ± 17.17	[86.36, 91.21]	94.96	90.56–96.42
Dice WT (%)	91.37 ± 7.17	[90.31, 92.33]	93.39	90.37–95.76
Dice Mean (%)	88.23 ± 9.70	[86.75, 89.54]	91.85	85.44–94.11
HD95 ET (mm)	4.09 ± 12.35	[2.52, 6.13]	1.41	1.00–2.24
HD95 TC (mm)	3.93 ± 8.17	[2.87, 5.21]	1.41	1.00–2.24
HD95 WT (mm)	3.74 ± 5.99	[3.01, 4.71]	2.24	1.41–4.00
HD95 Mean (mm)	3.98 ± 6.24	[3.16, 4.95]	1.91	1.33–3.35

SD, standard deviation; CI, confidence interval; IQR, interquartile range.

## Appendix B

To provide additional evidence for the training stability of PF-CMNet, we recorded the learning curves during the main full-modality training process. As shown in Figure A1, the training loss, validation loss, validation Dice, and validation HD95 were monitored across epochs. The curves show that the model achieved stable convergence, with decreasing training and validation losses, increasing validation Dice, and gradually stabilized validation HD95.
